# ERASing Confounders: Measuring Differences in Post Operative Pathways

**DOI:** 10.1177/22925503241268888

**Published:** 2024-08-01

**Authors:** Cameron F. Leveille, Brian Chin, Christopher J. Coroneos

**Affiliations:** 1 Division of Plastic Surgery, Department of Surgery, 3710 McMaster University, Hamilton, ON, Canada; 2 Department of Health Research Evidence and Impact, 3710 McMaster University, Hamilton, ON, Canada

The adoption of Enhanced Recovery After Surgery (ERAS) protocols in immediate deep inferior epigastric perforator (DIEP) flap breast reconstruction aims to optimize perioperative care, reduce the length of hospital stay, and minimize opioid use.^1,2^ Studies investigating ERAS protocols in DIEP flap breast reconstruction have employed either a case-series,^3-5^ comparative designs analyzing patient outcomes between cohorts of hospital centres^6,7^ or historical controls before and after the implementation of ERAS guidelines.^8-10^ Despite the promising results from studies, the existing literature has its limitations as studies are predominantly retrospective in nature.

The authors performed a retrospective review of the ERAS protocol in patients undergoing breast reconstruction during the COVID-19 pandemic, with the primary outcomes being hospital length of stay and opioid use.^11^ The authors concluded that implementing an ERAS protocol showed a significant reduction in hospital length of stay and opioid consumption without increasing adverse events. Although showing promising results, there are several inherent limitations in this before-and-after ERAS implementation study design that may have led to an inflated effect size. First, comparison of the ERAS cohort with a historical control can introduce confounding factors like changes in surgical technique, general trends in postoperative management, or provider experience over time contributing to a learning curve. Second, the ERAS group received intraoperative regional nerve blocks, which facilitated opioid-sparing analgesia. While regional blocks have become recommended criteria of ERAS protocols,^12^ the significant decreased abdominal wall morbidity from the transabdominal plane block have demonstrated to be the main driver in decreasing opioid consumption^13^ and is consistent with the changes seen in this study.^9,12^ Furthermore, the authors excluded patients who did not proceed with the ERAS pathway in the second cohort which may have introduced selection bias. Finally, the non-ERAS control group appeared to have a higher baseline complication rate compared to the ERAS group since ERAS helps to reduce complications (e.g. deep vein thrombosis and pulmonary embolism) by the virtue of earlier mobilization.^8^ As such, if the authors compared patients without complications between the two groups, the impact of ERAS protocols on outcomes like length of stay may be more modest or similar between cohorts.

The authors could have employed various strategies to mitigate these limitations. One such strategy is analyzing the outcomes in a contemporaneous cohort receiving ERAS without nerve blocks to isolate the impact of the full ERAS protocol independent of regional anesthesia techniques. Another strategy would be performing a subgroup analysis comparing only patients without complications between the ERAS and non-ERAS groups to evaluate if length of stay differences persisted after accounting for baseline complication rates. While the reported study provides valuable evidence for ERAS in DIEP reconstruction, addressing these methodological considerations could strengthen the findings and better elucidate the true impact of standardized ERAS pathways in this patient population. Exploratory analyses are needed to determine which components of the ERAS protocol in DIEP reconstruction contribute to each of the outcomes examined in the study, or if in fact, the whole ERAS protocol is more than a sum of its parts.
